# Extracts from Proceedings of the Indiana State Dental Association

**Published:** 1868-10

**Authors:** 


### Proceedings of Societies.

### EXTRACTS FROM PROCEEDINGS OF THE INDIANA STATE DENTAL ASSOCIATION.

TENTH ANNUAL MEETING JUNE 30TH, 1868.

*First Day.*

FILLING TEETH.

Dr. Hunt described his method of filling approximal cavities, which he stated was nothing new, and was not different from the general practice of good operators. He could work almost any gold in the market, and did not think there was so much difference in the preparations of gold, as is claimed by some. He inquired as to the experience of members present with plastic gold, and stated that he had tried Lamm's but thought it too uncertain in its preparation, and not reliable.

Dr. Rawls spoke of restoring the natural contour of the teeth, and of the retention of foreign substances between them.

Dr. Watt said he did not feel like going into the merits of the subject; but plastic gold was the material to which we would some day have to look for the best results in filling teeth. The preparations in market are simply precipitations of gold; and various precipitants are employed. These precipitated golds can be produced in the market as cheap, or cheaper than the foils, but crystalized gold can not be afforded near so low; and uniformity of texture can not yet be attained in all cases, with any plastic gold.

Dr. Taft referred to the methods of filling, and restoring the contour of decayed teeth, and thought it best in all cases to restore the natural shape of the tooth as nearly as possible. To secure success you must have good material, good instruments, and, above all, be a good operator. He can not at all times operate equally well; and it is best at sometimes to decline operating for the time being, on this account. Does not leave his gold exposed to the air and dust, but keeps it sealed up, and does not touch it with his fingers. Gold handled with the hands will smoke in the flame of the lamp, and can not again be entirely freed from debris, which will hinder its welding. To handle the gold, take a towel or large napkin properly folded in six or eight thicknesses, and roll the gold in it. (The manner was shown.) Does not bring the gold to a red heat in annealing—a very important matter and worthy of attention. Gold is not always uniform, and the manufacturers themselves can not tell why. He had often met with gold that did not work well, and that he would not use for filling teeth.

Dr. Hunt said he could easily see how an operator that depends entirely on the adhesive property of gold can not work all kinds of gold, but he does not depend on this property in all cases.

Dr. Richardson said his method was very simple and uniform. He uses foil and crystal gold. Is not partial to contour fillings, and is satisfied that in many instances it is not best to restore the original contour of the teeth. Would not build out on a frail wall, and prefers to mutilate a tooth a little for the sake of a good operation for service. Would in all cases have a free separation of the molars and bicuspids, so as to give room to work and finish. He is a little skeptical on the subject of crystal gold, not from his own experience, but from reports of others. Takes more time for a filling of Watt's gold, than with foil. Would like to hear something as to the relative value of foil and crystal gold.

Dr. Taft wants space to operate, but gets it mostly by wedging, which, however, will not give it in all cases, but it will keep the gum and moisture out of the way. Passes the common separating file between the teeth, and has the walls firm and strong; then he mortices down from the crown with chisels, or other instruments. He sometimes uses a broad wedge to build against, and fills as a common crown filling.

Dr. Richardson thought he might be misapprehended. He does not object to cutting away the crown of the tooth for anchorage, &c., but relies upon it. He cuts away till he gets good, strong marginal walls, and only fills out to the free surface, be it V shaped or not.

Dr. M. Wells said he was so unfortunate as to have a V shaped space between teeth in his own mouth, to his great annoyance, and would prefer to have it closed up.

Dr. Moore wished to know if teeth that have been separated, and closed up again, should be separated with the file.

Dr. Taft said the use of the tooth-pick would, in most cases, prevent the difficulty, but if the tooth is moveable and closes up, separate freely, and if necessary repeat the operation.

Dr. Stanley makes the surface of the filling convex, and does not have much trouble by the closure of the teeth.

President Goode wished to hear from the Association. He wants light. Would not file some teeth to separate them; rather extract a tooth to give room. He has trouble from crumbling with plastic gold, but likes crystal gold.

Dr. Richardson would like to know if the several forms of crystal gold are reliable for filling teeth if properly manufactured.

Dr. Watt replied that they are perfectly so. *Crystal gold* is as pure as any form of gold, and will weld more readily than foil. Fillings are now good that were made when it was first introduced, in 1853, '54 and '55, even with very defective instruments. The failures were not all failures of the gold, but of the operators. Some of the best operators use it exclusively, and it is of no use to deny its utility. We might as well condemn water as a medium to swim in, because some go to the bottom.

Dr. Morrill moved that a committee of three be appointed to take proper notice of the death of Dr. D. M. Weld, late of Terre Haute. Carried unanimously. The committee was constituted as follows: Joseph Richardson, Chairman, W. F. Morrill, A. M. Moore.

### ANÆSTHETICS.

Dr. Purcell spoke of Nitrous Oxyd, and has made some very good improvements in his apparatus for manufacturing the gas. He said it should be made slow and regular. Sprague's apparatus is the best. The gas-holder should be close to the patient, and the tubes large, free and short. He objects to the zinc holder, and the attachment of the inhaling tube to the top of the holder, and prefers galvanized iron, and a connection from the bottom.

Dr. Richardson uses Sprague's apparatus, without the regulator, which is like the play of Hamlet with Hamlet left out; but he cannot get the regulator to work well. He likes the gas for very brief operations, but after the first administration, it has not succeeded well with him at the same sitting; cannot give it in cases where many teeth are to be taken out with very good success.

Dr. Morrill spoke of Sprague's apparatus, and the manner of remedying the trouble alluded to by Dr. Richardson. He succeeds better with the gas after the first administration, at the same sitting. The second and third times the patient does better—learns how to breathe it, and he rarely finds a case in which he can not effect anæsthesia. He spoke of some extracting, any way to get the tooth out in a hurry, lacerating the gums, etc., and the practice was severely condemned. Thinks the nitrous oxyd, the greatest aid of anything yet in the extraction of teeth.

Dr. Stanley likes the gas very much, uses $\frac{3}{4}$ inch tube, but often takes off two of the three weights that balance the holder, to facilitate the supply to the patient.

Dr. Morrison, (Noblesville) has used the gas about two years, likes it well, but does not succeed so well with it the second administration at the same sitting.

Dr. Purcell thinks that no patient should have more than from four to six teeth extracted at any one time, or on the same day, and that a repetition of the gas is seldom necessary. Can not, in any case, succeed so well the second and third administrations, and generally fails the third. Prefers to wait one, two or three days. Never refuses a patient on account of heart disease, or other cause. One woman brought her friends along to see her die. Gave her the gas four times—extracted thirteen teeth, and she yet lives. Gave it twelve minutes for a surgical operation that was quite severe. The valve was opened and closed alternately, and air allowed to enter.

Dr. Goode does not approve of gas in heart disease, but has seen it given without serious results. Gives it through a long tube, but thinks it objectionable.

Dr. Richardson said we talk familiarly about heart disease, but it does not mean anything. There are a great many diseases of the heart, and we ought to know the pathological condition of this organ better. We will have to go further in this direction in our investigations. Gas is an excitant, and in certain pathological conditions, that we can conceive of, it would not be admissible, as in valvular disease. He does not give chloroform at all, on his own responsibility, and thinks the gas should not be given indiscriminately. We need a little curbing in this respect.

Dr. Watt said the world is indebted to the Dental profession for the discovery of anæsthesia, and for the principal researches in this direction. The medical profession does not seem so much interested. Pain, and the sense of feeling are not the same. Anæsthesia suspends the functions by which pain is felt. It is not necessary that we should be unconscious, that we feel no pain. Less than a year ago he began a series of experiments on himself and others. Chlorine was added to the gas, and the effects on the system noted. The complexion became "ashey" and remained so for days in succession. (Many other experiments were made, but the account of them could not be noted by the Secretary.) Binoxyd of Nitrogen is the most common impurity in the gas. Of this, the chemical reactions and changes, in air and vapor of water, were given, and the formation of nitrous and nitric acids from it, and their effects on the air passages, and the system generally were explained at some length.

This gas does not supercede the necessity for chloroform or ether. The vapor of ether is 2½ times as heavy as the air, and remains in the lungs to obstruct respiration; that of chloroform is still heavier, and the protoxyd of nitrogen is a heavy gas, and subjects the patient to the same difficulty. Respiration is not solely to get oxygen into the blood; but carbonic acid and vapor of water must be got rid of.

Nitrous oxyd is not breathed as air, but according to its own law. The respiration becomes slow in a minute or two, and may get down to three respirations per minute. Excess of carbonic acid in the air cells causes the buzzing in the ears; and the rapid expiration, sometimes noticed is to expel this carbonic acid.

The chemical action of nitrous oxyd on the blood was here given. More carbonic acid is given off at first than afterward; so to get rid of this excess, let the patient breathe air and gas alternately for a few moments. All the muscles of respiration should be perfectly free, and act well their part; there should be no tight dressing, and the position should be easy in all respects. The first effect of the gas is hyperæsthetic. As to inhalers and tubes, the less apparatus the better. The respiration through the tube should be as free as in the open air. The holder should be perfectly balanced, and smoothly hung, then have small weights, up to four or five pounds to balance for weak patients. The primary action is not on the heart, and the pulse does not vary under its influence. It was at 72 in self for an hour. To keep up the influence air is very important. Throw the valve open for a few inspirations, then breathe the gas again. The gas is a destroyer of the red corpuscles, and, therefore, not desirable for prolonged operations. The regulator of Dr. Sprague does not do away with the necessity for brains in the manufacture of the gas; but it does all that is claimed for it.

The committee on the death of Dr. Weld reported as follows:

Whereas, Almighty God has removed from our midst, by death, our worthy associate, Dr. D. M. Weld, therefore, be it

*Resolved*, That in the death of Dr. D. M. Weld, this Association has lost an esteemed and worthy associate and co-laborer, and that we deeply deplore this untimely loss of one so earnest and conscientious in the discharge of his duties, both as a practitioner, and in all the other relations of life.

*Resolved*, That a copy of these resolutions be entered upon the minutes of this Association, and that a copy be furnished to his family, and one to the DENTAL REGISTER for publication.

JOSEPH RICHARDSON, *Chairman.*
W. F. MORRILL,
A. M. MOORE.

### EXPOSED PULPS.

Dr. Taft now spoke of exposed pulps, and said that the great majority of Dentists destroy them; this is not the best plan. A living tooth is much better than a dead one. Pulps can not all be saved, dying pulps can not, but some can be, and even inflamed pulps can be treated and saved. Some thus treated years ago are still alive. Does not find many exposed pulps in his practice now. The method of treatment should be governed by the nature of the case. When we fill, all the space should be occupied, but the nerve must not be pressed. A non conductor must be used, as Hill's stopping, or os-artificial. The latter is not always the thing, without care in its application it will act as a caustic. Diseased pulps, if protruding, puncture and cover up, or treat therapeutically with creosote, glycerine and tannin. Puncturing or wounding is of no consequence, as to the safety of the pulp. Excision can be practiced, but is not very practicable.

Dr. Hunt has not had an experience so satisfactory, and has abandoned capping for some years back.

Dr. Morrison (Noblesville) was opposed to capping in the old way, which left a space not completely filled up, as this space will ultimately be filled by the pulp protruding through the orifice of exposure, and often cause its strangulation and death.

Dr. Taft fills with os-artificial in approximal cavities of the incisors, for some time, and afterward removes, and fills with gold. Sometimes the orifice closes up of itself.

Dr. Burgess feels relieved after hearing Dr. Taft; for five years ago, he gave his method substantially as that of the present, and was strongly opposed by the friends of arsenic.

Dr. Goode spoke of his old method, and a kind of heroic experiment with hot fusible metal, but has success now in the manner described by Dr. Taft, yet he thinks we need more light on the subject.

Dr. Hunt suggested that to know the work was well done, it must be undone and examined to prove the fact.

Dr. Watt said he had often undone the work, and proved the success. The chances for success are the same as in compound fracture, or gun-shot wound.

### OS-ARTIFICIAL.

Dr. Taft said he had been shown an os-artificial that he hoped soon to be supplied with.

Dr. Eddelman said he was not much conversant with the os-artificial referred to, but had a specimen, which was shown. It must be kept dry while being introduced, and has a better finish than the common os-artificial.

### ALVEOLAR ABSCESS.

Dr. M. Wells uses carbolic acid, &c., in fang.

Dr. Keightley related a case that was not cured by extraction.

Dr. Watt has used a compress to prevent opening externally, in cases of inferior bicuspids, and is very particular to have the opening on the inside. He resorts to hypodermic injections, before suppuration commences, with good success. Medicines administered in this way are twice as potent, and perhaps, four times as speedy in their operation, as when taken in the ordinary way. Uses solution of morphine, gr. 16 to oz. Tincture of opium is also used. The alcohol in the tr. opi'm. causes severe pain for a short time. A case was given to illustrate, with good results following. The advantages of opium over aconite and belladonna are in its acknowledged influence in subduing inflammation. In external treatment, sometimes uses spray to freeze the gum, then use trephine, and apply carbolic acid, &c., in opening.

Dr. Goode related a severe case treated with leeches, with good results.

Dr. Purcell related an extremely bad case that was treated in 1853 or '54, and is yet well. He uses creosote and iodine.

Dr. Rawls uses the same treatment externally.

Dr. Hancock related a case of a second bicuspid that opened externally, which was cured.

Dr. Morrison (Noblesville) gave a case of a central incisor that he filled with gold to the apex, then treated the abscess by cutting down upon it with an abscess lancet, and pressing a pledget of cotton saturated with creosote to the bottom of the opening. The case was successful.

### SENSITIVE DENTINE.

Dr. Hancock gave his experience with morphine. Fails in one out of three cases, as a local application. Sometimes uses it systematically, but uses it dry in cavity with best success.

Dr. Rawls uses solution of chloride of zinc.

Dr. Newby uses chloride of zinc and morphine, both often fail, and the spray has also failed in his hands.

On motion of Dr. Hunt the sum of ten dollars was voted the Young Men's Christian Association for the use of their rooms for the meetings of the Association.

On motion the thanks of the Association were tendered Drs. Watt, Taft and Keely for their kind participation in the deliberations of the Association.

On motion of Dr. Purcell, the subject of Dental Legislation was again taken up. The committee of last year was continued with discretionary powers, in accordance with Dr. Moore's resolution of yesterday.

Dr. Watt spoke of the Ohio law and gave us encouragement. He hoped that we would move in the matter before emigration commenced from his State, and that Indiana would not become the paradise of quacks.

Dr. Goode offered the following resolution which was unanimously adopted:

*Resolved*, That Professor George Watt be elected an honorary member of this Association, and as a further testimonial of out high appreciation of his invaluable services to this Association, and to the profession generally, we request him to accept the sum of one hundred dollars.

Dr. Watt then entertained the Association for some time in a kind of miscellaneous way. He first spoke of *Tangle Tent* (Laminaria Digitata) for Dental purposes. It was used in surgery, in fistulous openings; and as a dilator of the os-uteri. He thought it would be good for wedging, to separate the teeth. It expands three times its diameter, as seen in the specimen present, and has great force. It will do its work in about three hours.

Nitrous Oxyd, in many cases acts better the second and third administration; in others it does not. In some he would not give it a second time, but it does well in the majority of cases. Rules to govern in repeating: disposition of patient, breathing freely the first time, and absence of pallor, &c.

A case, of an operation on flexed knee was given, in which the gas was repeated a number of times. Ptyalism followed, and was attributed to the gas--needs to be tested further in this direction. The bile is a carbo-hydrogen, and may be removed from the blood by the gas. Jaundice may be thus cured. The therapeutic dose is from five to six gallons, slowly breathed. From six to eight doses, thus taken daily, or twice a day will produce ptyalism. To get its therapeutic effects, it must be given in doses as above, and not as an anæsthetic. It relieves excess of venereal passion—makes a beast of a man as modest as a maid; subdues the *passion* while the venereal *power* is not lost. Finds difficulty in these investigations on account of practicing a specialty. Physicians do not co-operate as they should. Wants it tried on females as well as males. More light is needed. Has given the gas in epilepsy and softening of the brain without bad results, and in this connection the duty of the profession to patients, and the difference between a profession and a trade were defined. Would not give the gas to over-heated or excited patients, as the red corpuscles will not take up the oxygen fast enough to cause anæsthesia. The menstrual period is unfavorable on account of hysterical excitement, and the loading of the blood with the debris of the period.

